# Mitotic progression and dual spindle formation caused by spindle association of de novo–formed microtubule-organizing centers in parthenogenetic embryos of *Drosophila ananassae*

**DOI:** 10.1093/genetics/iyac178

**Published:** 2022-12-14

**Authors:** Kazuyuki Hirai, Yoshihiro H Inoue, Muneo Matsuda

**Affiliations:** Department of Biology, Kyorin University School of Medicine, Mitaka, Tokyo 181-8611, Japan; Biomedical Research Center, Kyoto Institute of Technology, Kyoto, Kyoto 606-8585, Japan; Department of Biology, Kyorin University School of Medicine, Mitaka, Tokyo 181-8611, Japan

**Keywords:** acentrosomal spindle poles, diploidization, monastral bipolar spindles, parallel microtubule interactions, syncytial nuclear divisions

## Abstract

Facultative parthenogenesis occurs in many animal species that typically undergo sexual reproduction. In *Drosophila*, such development from unfertilized eggs involves diploidization after completion of meiosis, but the exact mechanism remains unclear. Here we used a laboratory stock of *Drosophila ananassae* that has been maintained parthenogenetically to cytologically examine the initial events of parthenogenesis. Specifically, we determined whether the requirements for centrosomes and diploidization that are essential for developmental success can be overcome. As a primal deviation from sexually reproducing (i.e. sexual) strains of the same species, free asters emerged from the de novo formation of centrosome-like structures in the cytosol of unfertilized eggs. Those microtubule-organizing centers had distinct roles in the earliest cycles of parthenogenetic embryos with respect to mitotic progression and arrangement of mitotic spindles. In the first cycle, an anastral bipolar spindle self-assembled around a haploid set of replicated chromosomes. Participation of at least one microtubule-organizing center in the spindle was necessary for mitotic progression into anaphase. In particular, the first mitosis involving a monastral bipolar spindle resulted in haploid daughter nuclei, one of which was associated with a microtubule-organizing center whereas the other was not. Remarkably, in the following cycle, biastral and anastral bipolar spindles formed that were frequently arranged in tandem by sharing an aster with bidirectional connections at their central poles. We propose that, for diploidization of haploid nuclei, unfertilized parthenogenetic embryos utilize dual spindles during the second mitosis, as occurs for the first mitosis in normal fertilized eggs.

## Introduction

Parthenogenesis refers to development from an ovum that was not previously stimulated or penetrated by a sperm. The capacity of unfertilized eggs to develop as diploid embryos involves important alterations to the basic constraints on accidental initiation of parthenogenesis in sexually reproducing species. During the evolution of sexual reproduction, parthenogenetic reproduction has arisen sporadically in certain but diverse taxa including *Drosophila* (Stalker 1954; Suomalainen 1962; Templeton 1983; Suomalainen et al. 1987; Normark 2003; Engelstädter 2008; Neaves and Baumann 2011; Markow 2013; Riparbelli et al. 2017; Galis and van Alphen 2020; Bell 2021).

In *Drosophila*, reproduction is essentially sexual, but the process of early embryonic development is highly adaptable to parthenogenesis (Templeton 1983; Markow 2013; Riparbelli et al. 2017; Avilés-Pagan and Orr-Weaver 2018; Lv et al. 2021). Egg activation occurs during passage through the oviduct, independently of sperm penetration in the uterus (Page and Orr-Weaver 1997; Heifetz et al. 2001). Following oviposition by virgin females of *Drosophila* species (Markow 2013), meiosis has been released from arrest in metaphase I and proceeds to completion (Doane 1960; Mahowald et al. 1983). In unfertilized eggs, all haploid meiotic products including both the presumptive female pronucleus and polar bodies are normally arrested in a mitotic, or more properly, a metaphase-like state of the first nuclear cycle, indicating that DNA replication and chromosome condensation take place but chromosome segregation does not (Gergely et al. 2000; Riparbelli et al. 2000; Pérez-Mongiovi et al. 2005; Gartenmann et al. 2020; Vazquez-Pianzola et al. 2022). Furthermore, early embryonic development occurs in a syncytium—that is, oocyte meiosis and early cleavage divisions of fertilized embryos occur without accompanying cytokinesis, resulting in an embryo with all cleavage nuclei and polar bodies within a common cytoplasm (Sullivan and Theurkauf 1995; Fogarty et al. 1997; Riparbelli et al. 2000; Fischer et al. 2004; Pérez-Mongiovi et al. 2005; Deshpande and Telley 2021). In fact, these features are associated with parthenogenesis from unfertilized eggs of some, if not all, *Drosophila* species (Stalker 1954; Sprackling 1960; Carson et al. 1969; Futch 1972; Carson 1973; Futch 1979; Templeton 1983; Riparbelli and Callaini 2003; Matsuda and Tobari 2004; Eisman and Kaufman 2007; Riparbelli and Callaini 2008; Chang et al. 2014), as well as gynogenesis (Fuyama 1986a, 1986b; Loppin et al. 2005) and androgenesis (Komma and Endow 1995) from fertilized eggs of *D*. *melanogaster*. In these examples of unisexual development with only maternal or paternal chromosomes, genetic evidence has been presented for patterns of diploidization by fusion between two haploid sets of chromosomes of meiotic products or cleavage nuclei, but it remains to be seen how two distinct sets of chromosomes are successfully gathered into close apposition in the syncytium.

Diploidization of haploid parental chromosome complements is a special feature of fertilized eggs. Zygotic diploid nuclei are generally formed during the mitotic phase of the first cleavage division in many sexually reproducing animal species (Kawamura 2001; Loppin and Karr 2005; Lindeman and Pelegri 2012; Reichmann et al. 2018; Rahman et al. 2020; Cavazza et al. 2021; Schneider et al. 2021). For early *Drosophila* embryos, the microtubule-organizing centers (MTOCs), centrosomes, are essential (Rothwell and Sullivan 1999; Basto et al. 2006; Stevens et al. 2007; Varmark et al. 2007; Rodrigues-Martins et al. 2008). In fertilized eggs, two centrosomes are newly constructed from template centrioles contributed by the sperm and maternally provided pericentriolar material (PCM) components including a number of proteins involved in microtubule nucleation, which compensate for the prior elimination of maternal centrosomes during oogenesis (Loppin et al. 2015; Blake-Hedges and Megraw 2019). The lack of centrosomes during meiosis, as well as the inhibition of de novo centrosome formation in unfertilized eggs, is speculated to be a mechanism to prevent parthenogenesis in *Drosophila* (Pimenta-Marques et al. 2016; Yamashita 2018).

Upon fertilization, the centrosomes are initially associated with the male pronucleus located deep within the egg and serve as the sperm aster, along which the distant female pronucleus is transported to close proximity of the male pronucleus in preparation for the first cleavage. The centrosomes then separate and form opposite poles of a mitotic spindle, organizing a microtubule array between them. Referred to as the gonomeric spindle, this first mitotic spindle consists of two halves of the bipolar microtubule arrays, each encompassing a parental set of replicated chromosomes. These microtubule units arranged in parallel are linked together at their distal ends, with an aster at the poles. Along the spindle, the haploid complements persist in separated groups at the equator, and then the two groups of separated chromatids become intermingled at the spindle poles, generating the zygotic diploid nuclei each associated with a centrosome (Guyénot and Naville 1929; Callaini and Riparbelli 1996; Komma and Endow 1997; Williams et al. 1997; Tirián et al. 2000; Loppin et al. 2015; Yamaki et al. 2016; Hirai et al. 2018). The nuclei undergo extremely rapid syncytial divisions, forming the blastoderm. In addition to the microtubule-organizing roles, centrosomes serve as hubs for the integration and coordination of other biological processes (Arquint et al. 2014; Ryniawec and Rogers 2021). In syncytial *Drosophila* embryos, centrosomes attached to spindle poles are implicated in the spatial control of Cyclin B destruction, regulating the exit from mitosis (Huang and Raff 1999; Wakefield et al. 2000). It should be noted that the first stages of embryonic development is entirely under maternal control through stored mRNA and proteins (Farrell and O'Farrell 2014; Yuan et al. 2016).

Centrosomes are also essential for parthenogenetic embryogenesis (Debec et al. 2010; Nabais et al. 2017). In many systems, including in *Drosophila*, the formation of centrosomes de novo has been extensively reported and in some cases mechanistically studied in vitro (Riparbelli et al. 2017; Pereira et al. 2021; Takumi and Kitagawa 2022). De novo formation of centrioles can be trigged in cases where cells lack all centrioles. Accordingly, MTOCs are self-organized in the cytosol of unfertilized parthenogenetic eggs of insects such as the viviparous pea aphid (Riparbelli et al. 2005), the hymenopteran *Nasonia vitripennis* (Tram and Sullivan 2000), and *Drosophila mercatorum* (Riparbelli and Callaini 2003; Eisman and Kaufman 2007; Riparbelli and Callaini 2008). In unfertilized embryos of parasitic wasps and honeybees, centrosomes are produced from oocyte nuclear envelope-derived cytoplasmic organelles with high concentrations of γ-tubulin (Ferree et al. 2006). In general, centrioles can be self-assembled without template centrioles, relying on only the concentration of components and PCM proteins (Pereira et al. 2021). Although new centrioles generally assemble in the vicinity of pre-existing centrioles in most proliferating cells, the centriole pair is not a completely essential component of centrosome formation (Martin and Akhmanova 2018). In syncytial embryos, the primary function of nucleus-associated centrosomes is to provide astral microtubules for proper nuclear spacing as cleavage divisions proceed (de Saint Phalle and Sullivan 1998; de-Carvalho et al. 2022). However, it remains to be seen whether the centrosomes are critical for the initiation of parthenogenetic development.

In *Drosophila* species, obligate parthenogenesis is known to occur only in one species *Drosophila mangabeirai*, whereas rare facultative parthenogenesis, referred to as tychoparthenogenesis, is far more common at least under laboratory conditions (Templeton 1983). One such species *Drosophila ananassae* provides unique experimental material with which to study the cytology of parthenogenetic embryos. Genetic variants that are associated with the ability to carry out parthenogenesis have been isolated from natural populations of otherwise sexually reproducing *D*. *ananassae* and its closely related species (Futch 1972, 1973; Matsuda and Tobari 2004). A previous study using a self-sustaining parthenogenetic strain showed that a causal gene maps to the left arm of chromosome *2* (Matsuda and Tobari 2004).

Identification of the exact means by which a diploid nucleus forms at the beginning of parthenogenetic development is crucial to elucidating the potential reproductive mechanisms. Here we report a detailed cellular analysis of meiosis and the first two cleavage divisions in unfertilized embryos produced by the parthenogenetic strain of *D*. *ananassae*, by comparison with those in unfertilized eggs and fertilized embryos produced by females of two sexual strains of the same species. The present study shows that, in parthenogenetic embryos, free MTOCs that are produced de novo in the cytosol play a central role in directing the earliest cleavage divisions and in delineating mitotic events by which diploidization can be achieved. In the egg, an anastral bipolar spindle arose from self-organization of microtubules around replicated chromosomes of a meiotic product. Progression of the first mitosis in the haploid state depended on incorporation of at least one cytoplasmic MTOC into the mitotic spindle. During the second division, parthenogenetic embryos often formed dual spindles, in which two separate spindles—each encompassing a single set of chromosomes—were arranged in tandem by sharing an MTOC at the connected spindle poles. Based on our cytological observations, we propose a model for the steps by which an essential diploid nucleus is generated in parthenogenetic embryos of *D*. *ananassae*.

## Materials and methods

### *D*. *ananassae* strains

The parthenogenetic stock, designated y-Im, was derived from flies caught in Taputimu, American Samoa (Futch 1972, 1973; Matsuda and Tobari 2004). It has been maintained in the laboratory for decades as a self-sustaining parthenogenetic line. The *X* chromosome of this strain is marked with *yellow* (*y*), a spontaneous mutation that occurred in the original stock. Gross morphology of ovaries and eggs appears normal. The obligate sexual strains used in the present study are AABBg1 and BKK17, which originated in Hawaii, USA and Bangkok, Thailand, respectively. Control embryos were essentially obtained from the AABBg1 stock, unless otherwise specified. Genetically heterozygous (F_1_ hybrid) females were produced as y^+^ females from the cross between y females of the parthenogenetic strain and wild-type males of the AABBg1 strain. *y* ^AM-203^ (abbreviated as *y* hereafter) and *white* (*w*) strains were used for the *X* chromosome segregation test. Stock maintenance and crosses were performed on standard cornmeal-glucose-yeast-agar medium at 24°C. All *D*. *ananassae* stocks were provided by Kyorin Fly, part of the National BioResource Project of Ministry of Education, Culture, Sports, Science, and Technology, Japan.

### Mitotic chromosome preparation

We made chromosome preparations with brains from third instar larvae using the air-dry method (Hirai et al. 2004), and chromosomes were stained with Giemsa. Squash preparations of embryonic chromosomes were made using embryos collected within 2 h after deposition as described (Vidwans et al. 2002), and chromosomes were visualized by DAPI staining. At least three sets of mitotic chromosomes in anterior, central, and posterior parts of individual embryos at the syncytial blastoderm stage were examined. Karyotypes were analyzable in ∼40% of the embryos in the sexual strain (*n* = 136), but in only <10% of embryos (*n* = 530) in the parthenogenetic strain, largely because of degraded or clumped DNA associated with developmental failure.

### Developmental assay

To examine the developmental progression of unfertilized eggs produced by females of the parthenogenetic strain, well-fed 6- to 10-day-old virgin females were allowed to lay eggs for 8 h on culture medium on which a drop of yeast had been placed (day 0). Every 25 (or fewer) laid eggs were transferred to the medium surface in a new vial. Then, at 48 h after being laid, the number of first instar larvae or post-hatching egg shells was scored. Individuals were scored through day 18. Eggs produced by mated and virgin females of the sexual strain AABBg1 and virgin females of F_1_ hybrids were examined in the same fashion.

To perform adult counts on a large scale, 10 females were allowed to lay eggs for 3 days on the culture medium. They were successively transferred to new vials every 3 days for additional three times and then were discarded. Adult offspring from each vial were scored on days 12 and 17 after the day of setup (day 0).

### *X* chromosome segregation test

To examine *X* chromosome segregation during female meiosis and earliest embryonic mitosis, *y*/*y* females were crossed to *w*/*Y* males. This test was initially carried out by single-pair matings, followed by mass matings using 10 females in each vial. After the establishment of the cross on day 0, parents were transferred to new vials on days 2, 4, 6, and 8 and were then discarded on day 10. The offspring were scored no later than day 18 after the females were allowed to lay eggs in each vial. Regular *X*-bearing eggs fertilized by *X*- and *Y*-bearing sperm yield *X*/*X* (+) female and *X*/*Y* (y) male offspring, respectively. Two classes of exceptional ova resulting from *X* chromosome nondisjunction or loss during meiosis are recoverable: diplo-*X* eggs fertilized by *Y*-bearing sperm and nullo-*X* eggs fertilized by *X*-bearing sperm, which give rise to *X*/*X*/*Y* (y) female and *0*/*X* (w) male offspring, respectively. *X*/*X*/*Y* (y) females are phenotypically indistinguishable from parthenogenetic *y* females. Exceptional *w* males can also arise from abnormal loss of the maternal *X* chromosome at the first mitosis of *y*/*w* zygotes. Loss of a paternal *X* chromosome results in *X*/*0* (y) males, which are indistinguishable from regular *X*/*Y* (y) males. The frequency of w males among offspring was calculated as [w males × 100]/[y^+^ w^+^ females + y males + w males].

### Collection of mature oocytes and embryos, immunostaining, and imaging

To examine meiotic spindles in mature oocytes, late-stage oocytes were separated from ovaries of 6- to 10-day-old females that were mated with males of the sexual strain and virgin females of the parthenogenetic strain and were treated as described (Takeo et al. 2010). Oocytes fixed with formaldehyde were stained for immunofluorescence with primary antibodies rat monoclonal anti-Tubulin (YL1/2, 1:300; Abcam) and rabbit anti-centrosomin (Cnn; 1:3,000; Lucas and Raff 2007), followed by secondary antibodies Alexa Fluor 488–conjugated goat anti-rat IgG (1:800; Thermo Fisher Scientific) and Cy3-conjugated AffiniPure goat anti-rabbit IgG (1:800; Jackson ImmunoResearch Laboratories), as well as the DNA dye DAPI.

Virgin or mated females were allowed to lay eggs on grape juice agar plates supplemented with fresh yeast paste. Embryos were collected within either one of the following time points after deposition: 5, 10, 20, 50, 80, 110, 140, 170, or 350 min. For a developmental assay using 3- to 6-h-old embryos, embryos laid within 3 h after deposition were collected and transferred to a humid chamber to develop at 24°C for another 2 h 50 min. Chemical dechorionation and vitelline membrane permeabilization were performed, taking 10 min for the processing before fixation and devitellinization in a mixture of methanol/heptane. Fixed embryos were stained for tubulin, Cnn, and DNA as described above and were also stained for Asterless (Asl) with guinea pig anti-Asl serum (1:3,000; Roque et al. 2012) followed by Alexa Fluor 647–conjugated goat anti-rat IgG (1:800; Thermo Fisher Scientific) as described (Hirai et al. 2018). Samples were mounted in Fluoro-KEEPER antifade reagent (Nacalai Tesque) and observed on a FLUOVIEW FV1000 with a 30× (1.05 NA) or 60× (1.30 NA) Sil UPlanSApo objective (Olympus). Images were acquired as *z*-series at 0.5-μm intervals with the laser power and gain settings determined for each embryo, and then were processed as maximum-intensity projections using ImageJ (NIH) and Adobe Photoshop 2022 (Adobe Systems).

### Data visualization

All data graphs were generated in Prism 7 (GraphPad Software).

## Results and discussion

### Parthenogenetic reproduction in *D*. *ananassae*

**Fig. 1.**
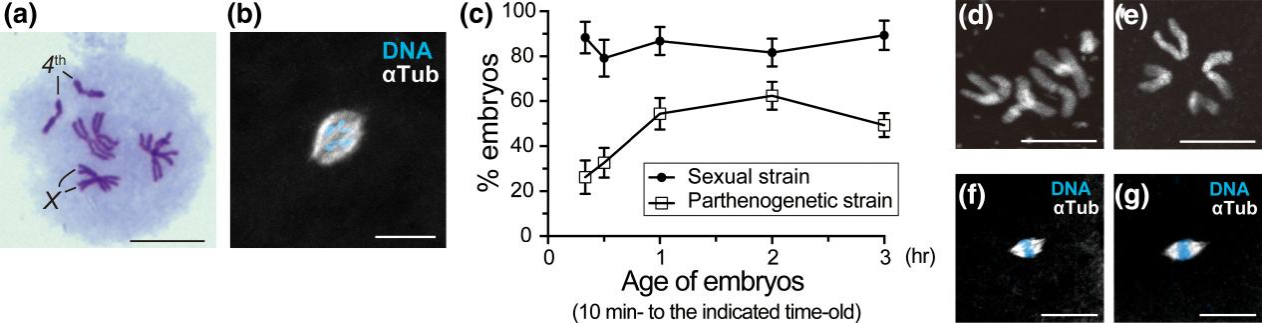
Development of parthenogenetic embryos. a) Parthenogenetic females are essentially *XX* diploids. The chromosome spread from the neuroblast of a female larva shows pairing of four pairs of homologous chromosomes, typical of normal *Drosophila* somatic cells. The *X* and fully heterochromatic fourth chromosomes are indicated. b) An unfertilized egg laid by the sexual strain AABBg1 was fixed with methanol and stained for microtubules (α-Tubulin, αTub) and DNA (DAPI). The two-color image on black background shows a bipolar array of microtubules self-assembled around condensed chromosomes of a meiotic product. The chromosome complement is inferred to be haploid from the number of chromosome arms; it is however ambiguous. c) The percentage of embryos that reached anaphase of the first cleavage or a more advanced stage. Embryos produced by mated females of the sexual strain and virgin females of the parthenogenetic strain were fixed within the time intervals from 10 min to the indicated time points (20, 30, 60, 120, and 180 min) after deposition and examined. The number of embryos analyzed ranged from 107 to 339 per time interval. Data are represented as the mean ± 95% confidence interval. d and e) Images of embryonic chromosomes. A diploid complement of chromosomes from the sexual strain (d) and a haploid complement of chromosomes from the parthenogenetic strain (e). f and g) Nonactivated mature oocytes were formaldehyde fixed, and stained as in (b). Females of both the sexual (f) and parthenogenetic (g) strains exhibited normal meiotic arrest at metaphase I with anastral bipolar spindles. Scale bars, 10 μm.

The self-sustaining parthenogenetic strain y-Im of *D*. *ananassae* is capable of virgin reproduction (geographic origin of the strain: Tutuila Island, American Samoa; Futch 1972). Female larvae that were examined from the stock were diploid for the *X* chromosome and three autosomes in neuroblasts, as expected for this species (Fig. 1a; *n* = 12; Kaufmann 1936; Marchetti et al. 2022). A large majority of adult individuals were females, but 0.3% (7/2,627) were males, which were considered to be sterile *X0* diploid individuals that resulted from the loss of an *X* chromosome during early mitosis (Futch 1972). In the context of haploid–diploid life cycle of animal reproduction, we would like to employ the ploidy values that indicate the number of sets of chromosomes as a multiple of the haploid genome, but not chromatin amount or DNA content that changes during the cell cycle, in this paper.

To determine the developmental stage critical for successful parthenogenesis that gives rise to adults, we successively counted the number of first instar larvae, pupae, and emergent adults that developed from unfertilized eggs laid by females of the strain. Unfertilized eggs laid by females developed into viable adults at a rate of 4.9% (Table 1). Clearly, the majority of embryos failed to hatch as larvae (Table 1). The extensive embryonic lethality indicates that a key process for successful developmental progression occurs during embryonic stages. It is also worth noting that, despite a complete lack of parthenogenetic capability in females of the sexual strain, parthenogenesis occurred in unfertilized eggs laid by virgin females of F_1_ hybrids (i.e. from matings between females of the parthenogenetic strain and males of the sexual strain), albeit in a very small percentage of cases (0.3%, Table 1). This genetic analysis argues that the parthenogenetic trait behaves recessively, and it is consistent with the idea of repression of parthenogenesis in the sexual strains of the species. In addition, given the weak effect elicited in F_1_ hybrids, we cannot rule out the other possibility that increased gene dosage confers the capacity of parthenogenesis.

**Table 1.** Development of eggs laid by females of the sexual and parthenogenetic strains of *D*. *ananassae*.

Developmental stage	Female parent			
	Mated females of sexual strain (*n* = 270)	Virgin females of sexual strain (*n* = 599)	Virgin females of parthenogenetic strain (*n* = 713)	Virgin females of F_1_ hybrids (*n* = 634)
First instar larvae (%)	88.5	0	8.7	1.1
Pupae (%)	83.3	0	5.3	0.3
Adults (%)	83.3	0	4.9	0.3

Entries indicate the percentage of laid eggs that developed until the indicated stages. The sexual strain AABBg1 and the parthenogenetic strain y-Im were used. Females of F_1_ hybrids were produced by crossing females of the parthenogenetic strain to males of the sexual strain.

To better understand chromosomal inheritance from mother to parthenogenetic offspring, we next examined chromosome segregation during meiosis and maternally driven earliest embryonic divisions by a conventional, highly sensitive genetic assay. By following the transmission of genetically marked *X* chromosomes in a cross, we were able to examine their behavior. Eggs that were aneuploid for the *X* chromosome were detectable; upon fertilization, those eggs were recoverable with different phenotypes among offspring. We could not analyze autosomes here. When *y*/*y* females from the parthenogenetic strain were crossed to *w*/*Y* males of the sexual strain, both sexual (+ females, y males, and w males) and parthenogenetic (y females) offspring were produced simultaneously (Table 2). w males are diagnostic of *X* chromosome misbehavior, as they result from exceptional nullo-*X* ova fertilized by *X*-bearing sperm. The females of the parthenogenetic strain produced w male offspring at a frequency of 0.3%, which was significantly higher than the <0.1% of w male offspring produced by those of the sexual strain (*χ* ^2^ = 14.59, d.f. = 1, *P* < 0.001). We note, however, that the level is fairly low. This result suggested that meiotic chromosome segregation errors are not a substantial factor with respect to the extensive embryonic lethality. Importantly, the sexual offspring obtained from the cross were diploids, but not phenotypically distinguishable triploids or intersexes (Gowen 1960). In addition, gynandromorphs, which would have resulted from abnormal mitotic loss of a single *X* chromosome from an *XX* individual at the first embryonic mitosis (Germani et al. 2018), were not detected from the cross. The absence of triploids and gynandromorphs was also the case for the parthenogenetic strain itself. To summarize, parthenogenetic strain females produced haploid eggs, and the accuracy of DNA replication and chromosome segregation is retained during both meiosis and maternally driven cleavage divisions in parthenogenetic embryos that survive to adulthood.

**Table 2.** Segregation data of the *X* chromosome.

	Offspring from *y*/*y* ♀ × *w*/*Y* ♂					Frequency of w ♂ (%)
Female parent	+ ♀	y ♀	y ♂	w ♂	Total	
Sexual strain	3,503	0	3,385	2	6,890	<0.1
Parthenogenetic strain	1,108	2,221	1,023	7	4,359	0.3

*y/y* females of the indicated strains were crossed to tester males carrying *w*, and offsprings were scored based on their phenotypes. Regular ova fertilized by *X*- and *Y*-bearing sperm resulted in + (*y*/*w*) females and y (*y*/*Y*) males, respectively. The w (*w*/0) males were descendants of exceptional nullo-*X* ova that resulted from nondisjunction or loss of the *X* chromosome during female meiosis, or they were produced by mitotic loss of the maternal *X* chromosome at the first cleavage division of *y*/*w* zygotes. Mitotic loss of the paternal *X* chromosome from *y*/*w* zygotes resulted in y (*y*/0) males that were phenotypically indistinguishable from *y*/*Y* males. y females arose from fertilization between exceptional diplo-*X* ova and regular *Y*-bearing sperm, as well as from parthenogenetic development of regular ova. Neither patroclinous w females, arising from androgenesis, nor gynandromorphs, arising from the loss of an *X* chromosome from an *XX* individual at the first embryonic mitosis, were detected from this cross. The frequency of w ♂ was calculated as an indication of *X* chromosome segregation errors (see Materials and methods).

### Bipolar spindle self-assembly around a haploid meiotic product and mitotic arrest in unfertilized eggs produced by females of *D*. *ananassae* sexual strains

To gain insight into the mechanical basis for successful parthenogenetic development, we next examined the behavior of chromosomes and microtubules cytologically during meiosis and the earliest cleavage divisions. Embryos were methanol-fixed and stained with an antibody against α-Tubulin and a DNA dye. We first confirmed that, in unfertilized eggs laid by females of the sexual strains AABBg1 derived from Hawaii (*n* = 30) and BKK17 derived from Bangkok (*n* = 9), meiosis had gone to completion and the eggs were arrested in a mitotic state of the first nuclear cycle. All four haploid complements appeared to be replicated and had undergone condensation. We found that, in the freshly laid unfertilized eggs, the chromosomes of the innermost meiotic product were surrounded by a microtubule array that often adopted a bipolar arrangement (Fig. 1b). Importantly, those unfertilized eggs showed terminal arrest in a metaphase-like state in the first mitosis but never entered anaphase with anastral spindles.

In contrast, in unfertilized eggs of the parthenogenetic strain, many, if not all, unfertilized eggs showed developmental progression into anaphase of the first mitosis or further. The proportion of such embryos was 26.2% in 10- to 20-min-old embryos, which gradually increased to a maximum of ∼50% in 10- to 120-min-old embryos. In the control embryos produced by mated females of the sexual strain AABBg1, the rates were consistently higher across all time intervals (Fig. 1c). The result suggests that development initiates slowly in parthenogenetic embryos compared with the sexual embryos. As there is overlap in the time ranges between collections of embryos, the rates of developing parthenogenetic embryos at certain time points from 20 to 120 min after deposition might be greater than those presented here.

Further development corresponding to formation of the syncytial blastoderm or beyond was evident among the 3- to 6 h-old parthenogenetic embryos (28.4%, *n* = 155; Supplementary Fig. 1b). The frequency was, however, much lower than that among the corresponding control embryos from the sexual strain (91.5%, *n* = 153; Supplementary Fig. 1a). An examination of karyotypes based on embryonic chromosome squashing revealed that all 61 syncytial blastoderm embryos from the sexual strain carried exclusively diploid chromosomal complements (eight chromosomes; Fig. 1d). However, the diploid class accounted for only 10% of parthenogenetic embryos (*n* = 39). The remaining classes were totally haploid (four chromosomes; 87%; Fig. 1e) or diploidy/haploidy mosaic (3%), representing embryos that generally died during embryogenesis. Based on these results, ∼3% of unfertilized eggs are assumed to become diploid syncytial embryos, which is on a similar scale to the low probability of parthenogenesis resulting in first instar larvae (8.7%; Table 1). However, the estimated rate of diploid parthenogenetic embryos was slightly inferior to the hutching rate. This may be attributable to the limited number of analyzable samples and/or development of mosaic embryos into diploid larvae. Taken together, the results indicate that at least two steps are rate limiting for successful parthenogenesis: (1) progression of the first nuclear division with a haploid set of chromosomes and (2) subsequent diploidization of haploid chromosome complements in the syncytium.

### De novo formation of MTOCs in the cytosol of the activated eggs produced by females of the parthenogenetic strain

**Fig. 2.**
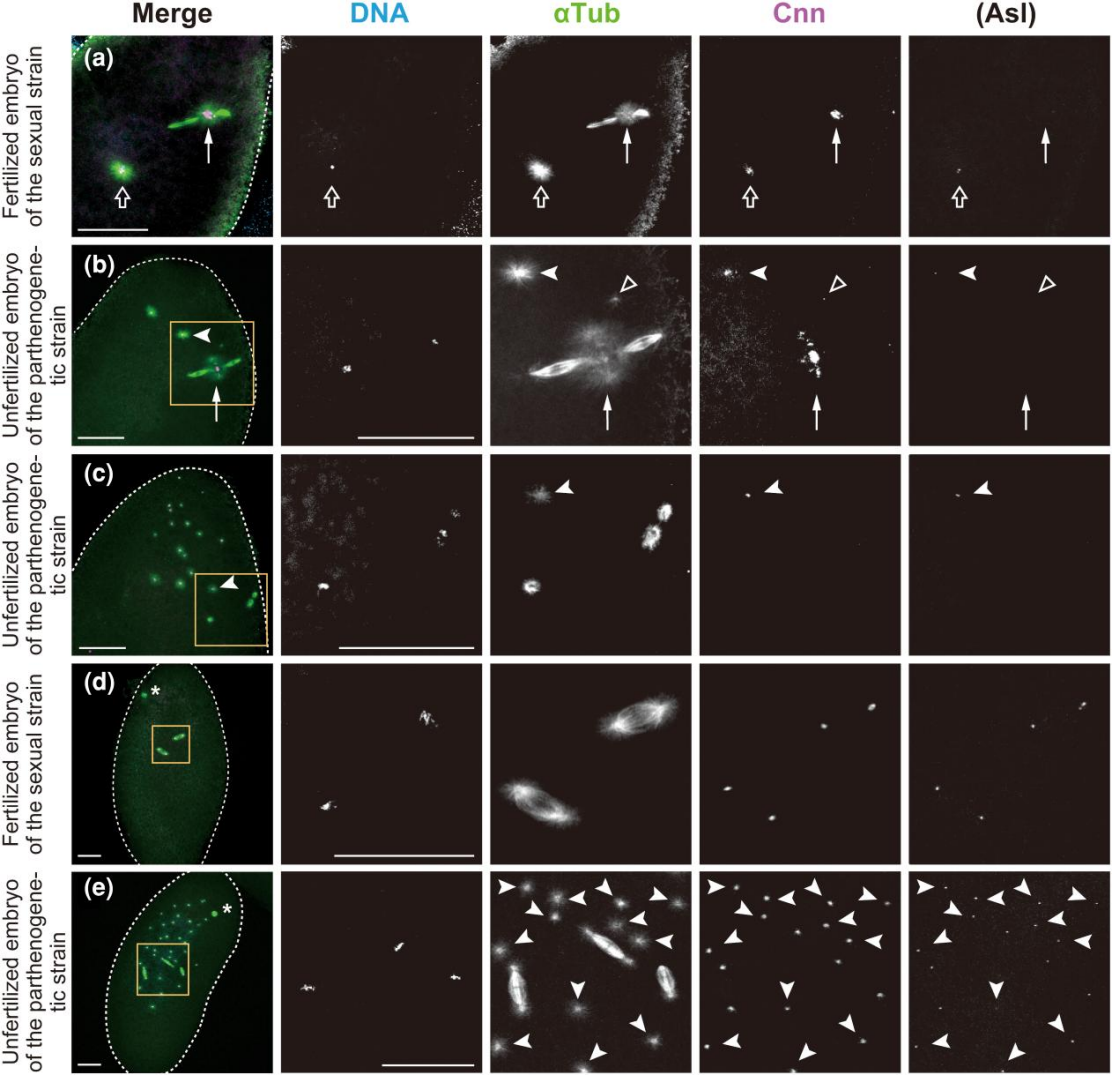
De novo formation of MTOCs in the cytosol of activated eggs produced by females of the parthenogenetic strain. Laid eggs and embryos were fixed and treated with antibodies against α-Tubulin (αTub) for microtubules and Cnn and Asl (not overlaid in merged images but only shown independently) for the PCM, as well as the DNA dye DAPI. In the merged images, the superimposed staining of microtubules and Cnn appears white. a and d) Fertilized embryos laid by females of the sexual strain AABBg1. b, c, and e) Unfertilized embryos laid by females of the parthenogenetic strain. The edge of each embryo is outlined with dashed lines for clarity. The boxed regions in the merged images in (b–e) are shown at higher magnification in the adjacent panels. a) The fertilized oocyte in metaphase II exhibits the meiotic aster located between the central poles of the two tandem spindles of the female meiotic apparatus (arrow) and the sperm aster in the center of the egg (outlined arrow). The former includes only Cnn but not Asl, whereas the latter includes both Cnn and Asl. Chromosomes on the spindles are not visible in the processed images. b) The unfertilized egg laid by the parthenogenetic strain shows two free asters, which are formed from MTOCs that include Cnn and Asl (one of them is indicated by a white arrowhead) at the anterior of the egg cytosol. A punctiform aster (outlined triangle) is detectable immediately adjacent to the prominent aster at the central poles of the meiotic spindles (arrow). c) After completion of meiosis, meiotic products are surrounded by arrays of microtubules. One is located centrally in the egg (on the lower left side of the boxed region), while the others lie near the cortex. Free asters of variable sizes are randomly distributed at the anterior of the egg. The arrow indicates one of the asters. d) The sexual embryo during the second mitosis shows synchronous mitotic progression with biastral bipolar spindles. The polar body lies near the cortex (asterisk). e) This parthenogenetic embryo contains a total of three mitotic spindles, in addition to the polar body (asterisk) and ∼30 free asters that are predominantly distributed in a region approximately one-half of the egg's length from the anterior end (arrowheads). One of the bipolar spindles is monastral, whereas the others are biastral. Ploidy level of the chromosome complements is not known. Scale bars, 50 μm.

We next addressed the question of how developmental progression can be induced in some, but not all, unfertilized eggs produced by the parthenogenetic strain. We first confirmed that there were no obvious differences in meiotic chromosome behavior and spindle morphology at all stages of meiosis between females of the sexual and parthenogenetic strains, consistent with the above-mentioned genetic results. Nonactivated mature oocytes in the ovaries exhibited normal metaphase I arrest. The chromosomes tightly massed at the equator of an anastral bipolar spindle in a region approximately one-third of the egg's length from the anterior end (Fig. 1f for sexual strain, *n* = 13; Fig. 1g for parthenogenetic strain, *n* = 11). In laid eggs, meiosis had resumed and progressed toward completion. The meiosis II spindles were composed of two small spindles in a tandem orientation (Fig. 2a from a female of the sexual strain; Fig. 2b from a female of the parthenogenetic strain). Although distal poles of the meiosis II spindles were anastral, a disk-shaped aster emerged transiently at the central poles. This MTOC was immunolabeled with the centrosome marker Cnn, which is localized extensively in the outer zone of the PCM, but was not labeled by another marker, Asl, whose localization is confined to the core centriole and inner zone of the PCM (arrows in Fig. 2, a and b; Lattao et al. 2017; Tillery et al. 2018). Shortly after completion of meiosis, meiotic spindles as well as the acentriolar aster disappeared normally in embryos produced by both parthenogenetic (Fig. 2c) and sexual strains, as in *D*. *melanogaster* embryos (Riparbelli and Callaini 1996; Cui et al. 2008).

A striking difference between eggs laid by females of the sexual and parthenogenetic strains was noticed in aster formation in the egg cytosol. In fertilized eggs of the sexual strain, the clearly visible sperm aster formed in the center of the egg (Fig. 2a, outlined arrow). Next to the sperm nucleus, the radial arrays of microtubules were nucleated by the MTOCs, which were labelled with both centrosome markers Cnn and Asl (Fig. 2a). In contrast, no asters were detected in unfertilized eggs of the sexual strains arrested in a mitotic state of the first cycle (*n* = 102 for AABBg1 eggs, 10 min–3 h after deposition; *n* = 136 for AABBg1 eggs, 3–6 h after deposition; and *n* = 52 for BKK17 eggs, 10 min–6 hafter deposition). However, in unfertilized eggs of the parthenogenetic strain, MTOCs with Cnn and Asl labeling (i.e. centrosome-like structures) formed particularly at the anterior of the earliest embryos, at a short distance from meiotic spindles and nuclei (Fig. 2b, arrowheads). We refer to the MTOCs that form spontaneously in the egg cytosol as “free MTOCs,” and “asters” as the microtubules emanating outward from any MTOCs. Those free MTOCs appeared as early as anaphase I (Supplementary Fig. 2a) and were detected in the majority of embryos laid by the parthenogenetic strain: 73.3% of eggs in anaphase I–telophase II (*n* = 30; Fig. 2, a and b) and >90% of post-meiotic embryos that were fixed 10–30 min after deposition (*n* = 292; Fig. 2, c and e). Nonactivated oocytes in prometaphase I–metaphase I completely lacked asters (Fig. 1g, *n* = 49). In summary, de novo formation of free MTOCs occurs after egg activation in the egg cytosol in the parthenogenetic strain.

Centrosomes duplicate precisely once per cell cycle during the canonical cell cycle (Callaini and Riparbelli 1990; Debec et al. 1996), but we noted that the number of free MTOCs varied across individual embryos. To assess de novo formation of MTOCs and their possible overduplication independently of nuclear divisions, we quantified free MTOCs over time in embryos showing no cleavage divisions beyond the first metaphase-like state. The number of free MTOCs observed was 6.7 ± 10.0, 10.1 ± 12.0, 10.7 ± 7.4, and 5.9 ± 4.9 (mean ± SD) in 10- to 30-min-old (*n* = 18), 10- to 60-min-old (*n* = 17), 10- to 90-min-old (*n* = 19), and 10- to 150-min-old (*n* = 15) embryos, respectively. An increase in the number of MTOCs over time was not evident, suggesting a transient production of MTOCs but not their self-propagation in the cytosol.

### Free MTOCs can be incorporated into the spindle for the first mitosis of parthenogenetic embryos

**Fig. 3.**
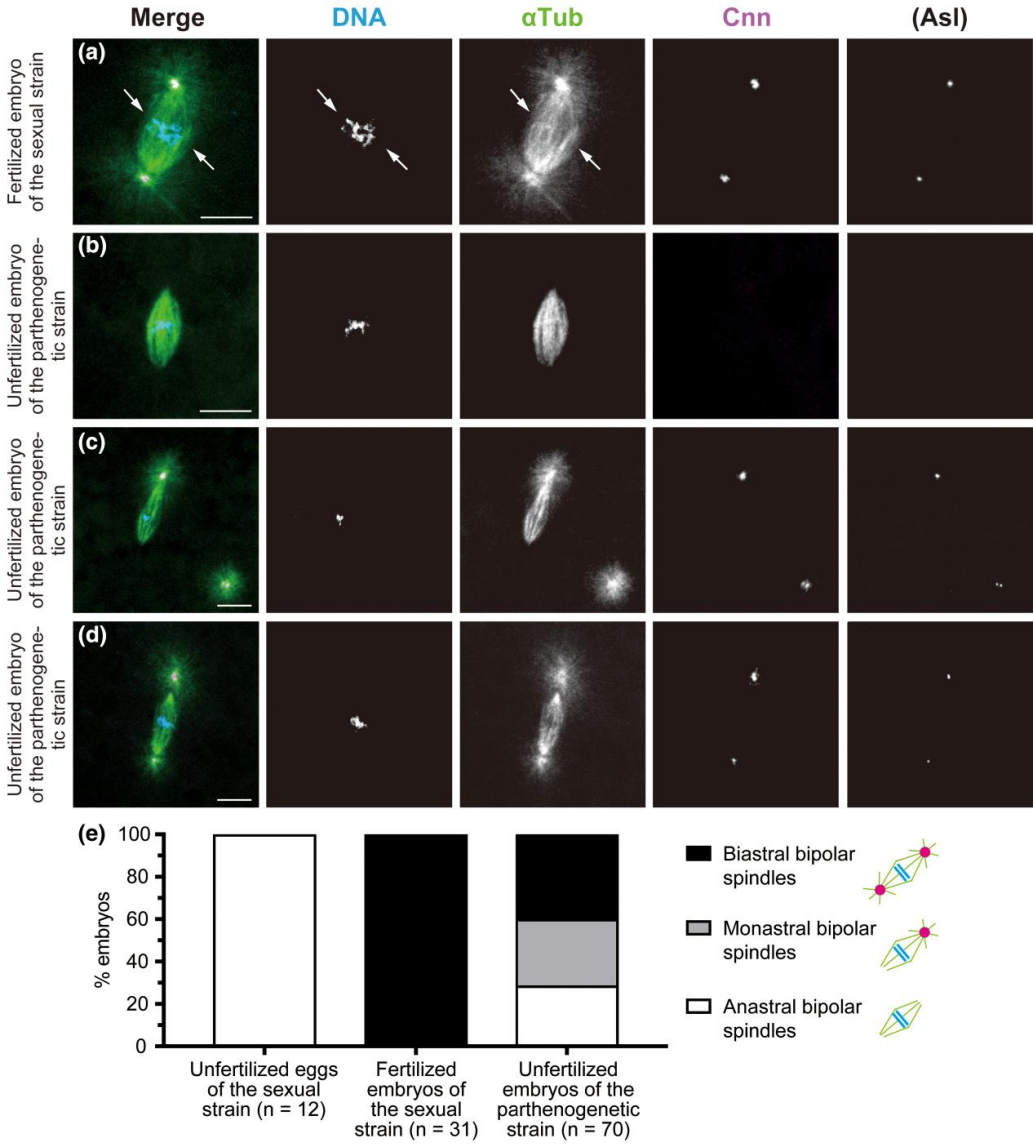
The first mitosis in sexual and parthenogenetic embryos. Laid embryos were fixed and treated with antibodies against α-Tubulin (αTub) for microtubules and Cnn and Asl (not overlaid in merged images but only shown independently) for the PCM, as well as the DNA dye DAPI. In the merged images, the superimposed staining of microtubules and Cnn appears white. a) In the fertilized egg produced by the sexual strain AABBg1, the first mitotic spindle is bipolar and biastral around two groups of replicated chromosomes derived from the ovum and sperm (arrows). b–d) In unfertilized embryos produced by the parthenogenetic strain, a set of replicated chromosomes, which is considered to be haploid, aligns at the equator during the first mitosis. Those spindles are smaller relative to the spindle in (a). Based on the number of asters associated with a bipolar spindle, spindles fall into three classes: (b) an anastral bipolar spindle that formed in an embryo containing four free asters in the cytosol (not visible in this image), (c) a monastral bipolar spindle and a neighboring free aster, and (d) a biastral bipolar spindle that formed in an embryo containing six free asters (not visible in this image). The asters at the opposite spindle poles vary markedly in size. e) The proportion of different forms of the first mitotic spindle. Scale bars, 10 μm.

Following the completion of meiosis and the pronuclear stage, syncytial embryonic development begins with a series of rapid nuclear divisions. To investigate the influence of free MTOCs particularly at the earliest stage of parthenogenetic development, we thoroughly examined the first mitosis using a collection of embryos fixed within 30 min after deposition. This developmental stage was assigned by the presence of a single mitotic spindle in individual embryos. In the control (sexually developing) embryos, a biastral bipolar spindle was assembled around parental sets of chromosomes in the interior of the embryo (Fig. 3a; *n* = 12). Typical of the first mitosis of the *Drosophila* zygote, the metaphase spindle appeared to be composed of two units of microtubule arrays each encompassing a haploid set of replicated chromosomes. In unfertilized embryos of the parthenogenetic strain, meiotic products were surrounded by microtubules, at least immediately after deposition (Fig. 2c). The embryos then self-assembled a bipolar spindle around the seemingly haploid set of chromosomes positioned in the interior, apart from polar bodies lying near the cortex (Fig. 3, b–d). Clearly, the spindles formed in parthenogenetic embryos were smaller (Fig. 3, b–d) relative to those formed around parental sets of chromosomes in sexual embryos (Fig. 3a), indicative of the former's haploidy (Komma and Endow 1995, 1997; Brent et al. 2000). Nonetheless, the predicted ploidy level is ambiguous.

Although the spindles similarly self-assembled around a meiotic product located centrally in unfertilized eggs of the sexual strain, they were invariably anastral (Fig. 1b); this was not always the case in parthenogenetic embryos. Allocation of asters associated with a bipolar spindle of the first mitosis differed substantially between individuals (*n* = 70); the spindles were anastral (28.5%; Fig. 3b), monastral (31.4%; Fig. 3c), or biastral (40.0%; Fig. 3d). The presence of monastral spindles containing one astral and one anastral pole (Fig. 3c), as well as a clear difference in the sizes of asters on individual biastral spindles (Fig. 3d), suggests that free MTOCs produced in the cytosol could coalesce into either one or both anastral poles of a bipolar spindle. We occasionally observed earlier association of MTOCs with microtubules that accumulated in the vicinity of individual chromosomes, but not the whole set of chromosomes (Supplementary Fig. 2, b and c). The consequence of such an occurrence is not, however, known.

### Mitotic progression occurs with astral, but not anastral, bipolar spindles during the first mitosis of parthenogenetic embryos

**Fig. 4.**
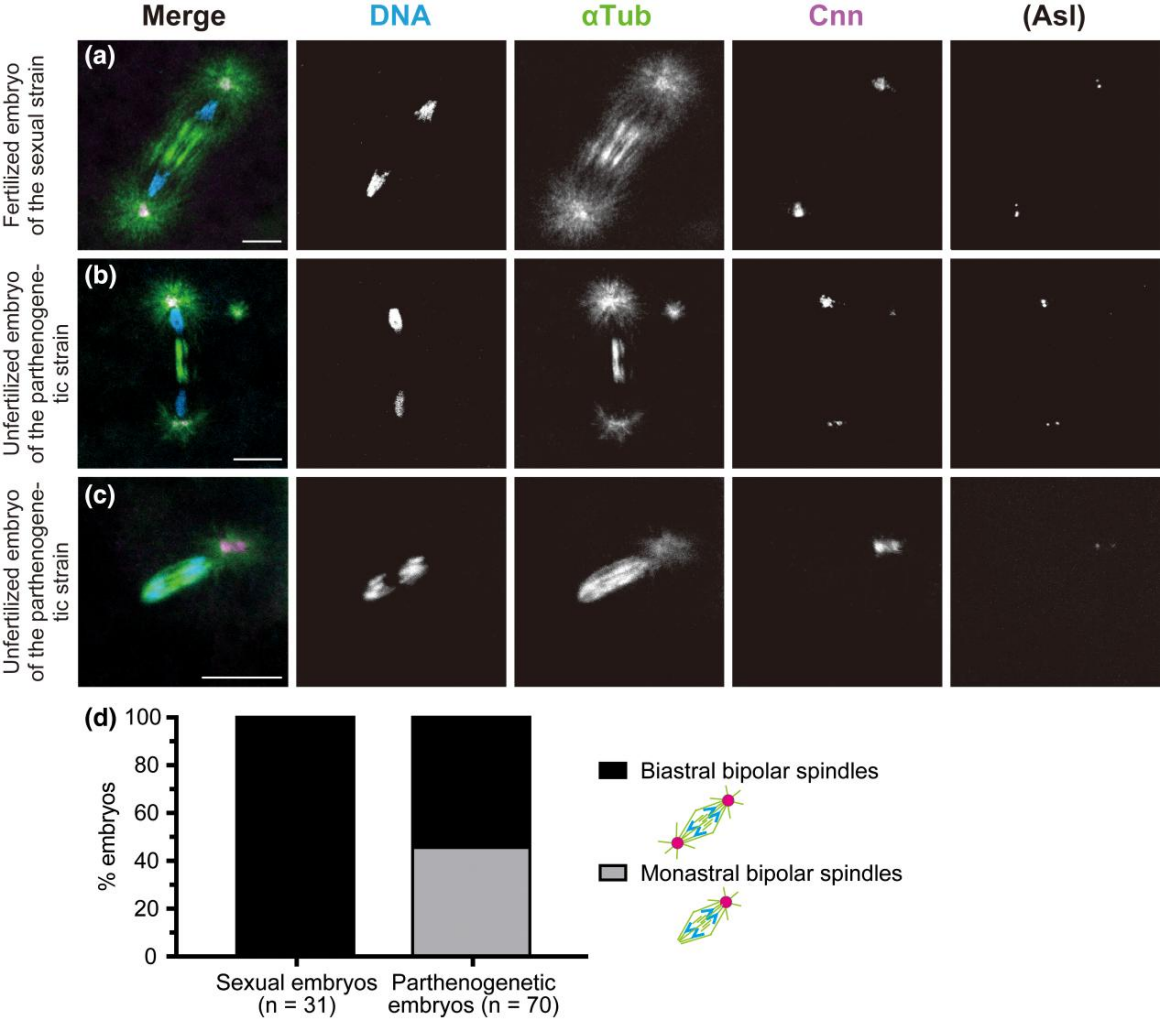
Chromosome segregation occurs on astral, but not anastral, bipolar spindles during the first mitosis of parthenogenetic embryos. Embryos laid by females of the parthenogenetic strain were fixed and treated with antibodies against α-Tubulin (αTub) for microtubules and Cnn and Asl (not overlaid in merged images but only shown independently) for the PCM, as well as the DNA dye DAPI. In the merged images, the superimposed staining of microtubules and Cnn appears white. a) During late anaphase of the first mitosis in a sexually developing embryo of the sexual strain AABBg1, chromosomes of maternal and paternal origin are gathered into a single mass as separated chromatids synchronously migrate toward the poles of the biastral bipolar spindle. b and c) Mitotic progression during the first mitosis of parthenogenetic embryos. The spindles are smaller relative to those formed in the sexual embryo (a). b) Late anaphase on the biastral bipolar spindle of the first mitosis in unfertilized embryos of the parthenogenetic strain. The upper chromosome set looks brighter than the lower one because of the difference in depth resulting from the tilted axis of the division. This is true for the microtubule-organizing centers of the nuclei and asters. One of the six free asters in the cytosol is shown in the upper right region of the image. c) Early anaphase on the monastral bipolar spindle of the first mitosis in unfertilized embryos produced by females of the parthenogenetic strain. The embryo contains one free aster (not visible in this image). d) Distribution of different forms of bipolar spindles during anaphase–telophase of the first mitosis. Note that no anastral spindle showed mitotic progression into anaphase. Scale bars, 10 μm.

To see the effect of MTOCs associated with the first mitotic spindle on mitotic progression, we then examined embryos in the subsequent anaphase and telophase stages. Mitotic progression into anaphase is heralded by the synchronous movement of two sets of separated chromatids away from the metaphase plate to opposite spindle poles, where the chromosomes are packaged into new daughter nuclei during telophase. In the control sexual embryos, all examined anaphase–telophase spindles of the first mitosis were biastral and bipolar (Fig. 4a; *n* = 31). However, in embryos produced by the parthenogenetic strain, anaphase and telophase figures were detectable on bipolar spindles that were not only biastral (Fig. 4b) but also monastral (Fig. 4c). These embryos exhibited orderly segregation of chromosomes. No anastral spindles showed progression into anaphase in embryos from the parthenogenetic strain (Fig. 4d; *n* = 70), despite their presence during the prior metaphase (Fig. 3, b and e). It should be emphasized that the presence of one MTOC at a bipolar spindle is both necessary and sufficient to confer mitotic progression.

The ratio of monastral to biastral spindles observed during anaphase–telophase (biastral, 54.3%; monastral, 45.7%) mirrors that during the prior stages (biastral, 56.0%; monastral, 44.0%; *n* = 50 from Fig. 3e). This suggests that, during the first mitosis of parthenogenetic embryos, astral bipolar spindles do indeed segregate the chromosomes, regardless of whether they are biastral or monastral. In contrast, mitosis in the parthenogenetic strain embryos terminated in a metaphase-like state without transition to anaphase with bipolar spindles devoid of any associated MTOCs. Such a mitotic arrest with anastral bipolar spindles is reminiscent of that which normally occurs in laid unfertilized eggs of sexual strains (Fig. 1b). Obviously, with MTOCs that have been produced de novo in the cytosol of eggs from the parthenogenetic strain, engagement of at least one of the free MTOCs in the first mitotic spindle is required to exit from the metaphase state. More specifically, in parthenogenetic embryos, the MTOC is not required for organizing a metaphase spindle around replicated chromosomes of a meiotic product, but association of a focused pole of the self-assembled bipolar spindle with an MTOC is crucial for the initiation of anaphase.

This notion is plausible because a similar mitotic arrest phenotype has been reported in *Drosophila* embryos defective in centrosome localization at spindle poles. Mutations in *D*. *melanogaster Cyclin B3* (Yuan and O’Farrell 2015; Garrido et al. 2020) and *Elys* (Hirai et al. 2018) exhibit maternal-effect lethality during a metaphase-like state of the first mitosis, accompanied by an anastral spindle that results from centrosome detachment from the spindle poles. Moreover, in fertilized eggs that are defective in pronuclear migration, an anastral spindle is assembled around replicated chromosomes of the female pronucleus, at a distance from the male pronucleus, where mitotic progression is halted in a metaphase-like state (Llamazares et al. 1999; Gergely et al. 2000; Riparbelli et al. 2000; Dix and Raff 2007; Varmark et al. 2007; Vazquez-Pianzola et al. 2022). We suggest that the MTOCs at the spindle poles regulate the mechanism of the spindle assembly checkpoint. In *D*. *melanogaster* syncytial embryos, chromosome segregation is triggered by cohesin cleavage and downregulation of Cdk1 (Oliveira et al. 2010). Thus, it may be that, in parthenogenetic embryos of *D*. *ananassae*, an MTOC located at one of the poles of the first mitotic spindle can promote anaphase onset, setting the key biochemical events in motion within the entire spindle.

### Dual spindle formation during the second mitosis of parthenogenetic embryos by arrangement of two spindles in tandem

**Fig. 5.**
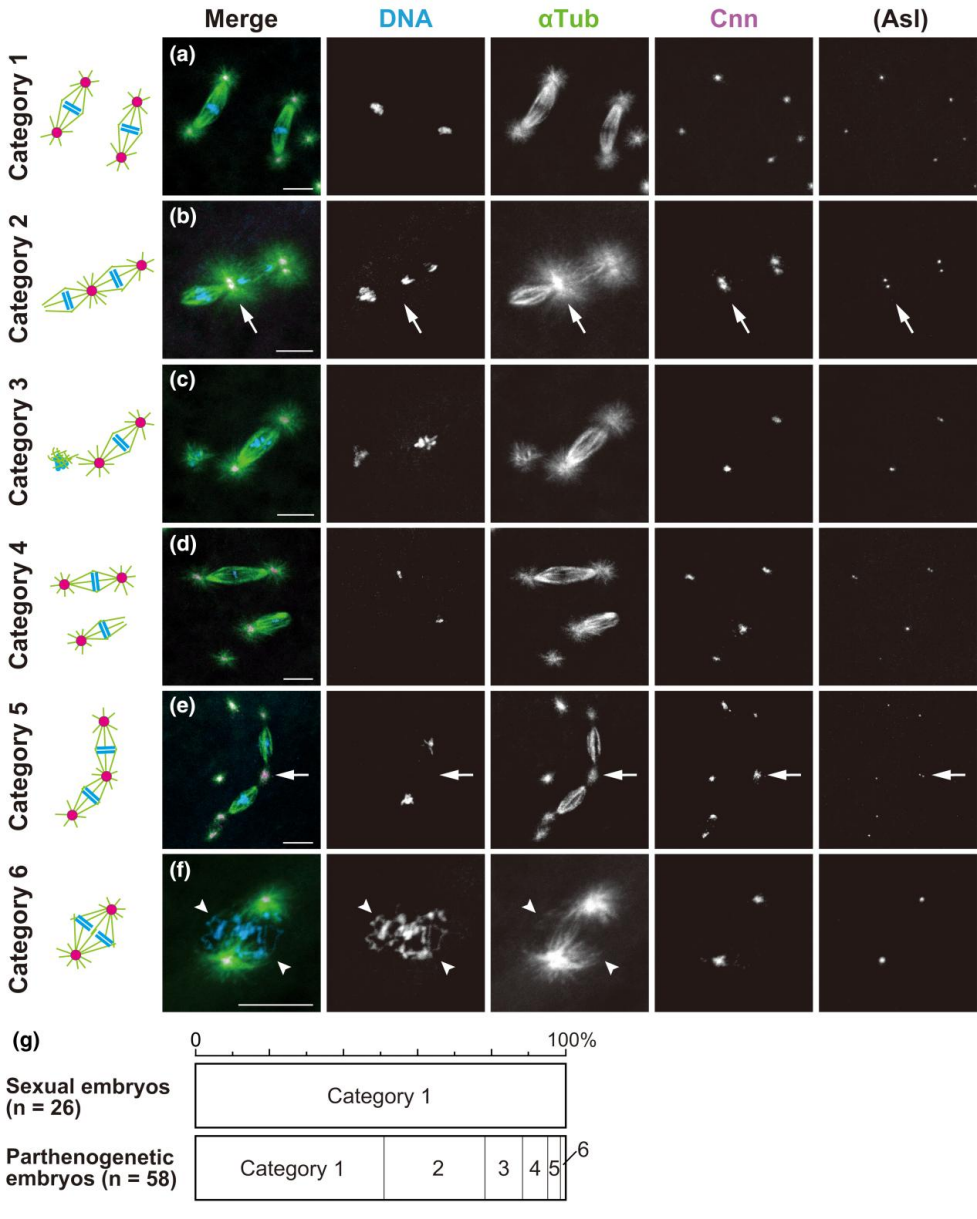
Dual spindles are formed by sharing an aster at the central poles during the second mitosis of parthenogenetic embryos. Laid embryos were fixed and treated with antibodies against α-Tubulin (αTub) for microtubules and Cnn and Asl (not overlaid in merged images but only shown independently) for the PCM, as well as the DNA dye DAPI. In the merged images, the superimposed staining of microtubules and Cnn appears white. Based on the configurations of their mitotic figures, embryos undergoing the second mitosis were assigned to the following six categories, listed in decreasing order of frequency. a) Category 1: two independent biastral spindles form. b) Category 2: two spindles form which are tandemly arranged by sharing an aster at the central pole (arrow). For the dual spindles, one of the distal poles is anastral whereas the other is astral. c) Category 3: a biastral spindle forms around one set of chromosomes and short arrays of microtubules form around the other set of chromosomes. d) Category 4: two independent spindles form that are biastral and monastral. For the monastral spindle, the astral pole appears more tapered than the anastral pole. e) Category 5: dual spindles form that are similar to those of category 2, but they differ only in that both distal poles of the dual spindle are astral in category 5. One of the distal poles (in the lower left) is associated with asters in pairs, whereas the other distal pole and the central poles (arrow) are associated with individual asters. f) Category 6: two units of bipolar arrays of microtubules form around the adjoining sets of chromosomes. The units are closely apposed, sharing asters at the spindle poles. The arrowheads mark the neighboring two sets of chromosomes on the spindle. g) The graph quantifies the six mitotic configuration categories formed during the second cleavage division. Scale bars, 10 μm.

We next wished to know what mechanism controls the second mitotic cycle with the daughter nuclei produced by the first cleavage division of parthenogenetic embryos. In the control (sexually developing) embryos, after DNA replication, progression of second mitosis was always synchronous and occurred with two independent, biastral bipolar spindles. The individual spindles were clearly separated but at a relatively close distance in the syncytium (Fig. 2d; *n* = 26). In parthenogenetic embryos, however, miscellaneous configurations of spindles were present around two sets of chromosomes that could be classified into six categories (Fig. 5; *n* = 59). It should be noted first that no parthenogenetic embryos contained two anastral spindles, which is consistent with the halting of mitotic progression on anastral bipolar spindles during the first mitosis, as mentioned above. About half of the embryos displayed an apparently normal pair of biastral bipolar spindles during the second mitosis (category 1, 50.8%; Fig. 5a). This frequency is in excellent agreement with that of the biastral bipolar spindles noted during the prior first anaphase–telophase stages (54.3%; Fig. 4d), lending support for the apparently normal nuclear and centrosomal cycles. We thus infer that most, if not all, category 1 embryos in the second cycle result from the first mitosis with a biastral bipolar spindle. Those embryos seem to undergo syncytial cleavage divisions in the haploid state (Supplementary Fig. 1b), resulting in lethality before hutching.

The remaining five categories of mitotic configurations, which were specific to parthenogenetic embryos, are presumed to have arisen from a first mitosis that was carried out on monastral bipolar spindles (Fig. 4c). Of the resultant daughter nuclei, which were considered to be haploid, one was associated with an MTOC and the other was not. Among the configurations of two spindles that were seen only in parthenogenetic embryos, a pair of tandemly arranged spindles was most common (category 2, 27.1% and category 5, 3.4%; Fig. 5, b and e, respectively). Those distinctive dual spindles consisted of two bipolar spindles that were connected at their central poles by sharing a single MTOC (Fig. 5, arrows), whereas the distal poles were kept completely apart. We assume that those dual spindles resulted from the coupling of neighboring biastral and anastral spindles through a shared MTOC during the second mitosis, as discussed below. In rare instances, a bipolar spindle formed around two distinct chromosome sets that were juxtaposed to each other (category 6, 1.7%; Fig. 5f). It is not known whether this configuration results from union of two independent bipolar spindles, or assembly of an all-inclusive bipolar spindle. It should be noted, however, that the spindle configuration of category 6 observed in parthenogenetic embryos bears a close resemblance to the normal first mitotic spindle in fertilized eggs of the sexual strain, which is composed of two units of microtubules around parental haploid sets of chromosomes (Fig. 3a).

In category 3 embryos, a biastral bipolar spindle assembled around one of the two chromosome sets, and the other chromosome set located nearby was surrounded by apparently short microtubules displaying random polarity with no aster association (10.2%; Fig. 5c). This situation probably reflected an early frame in the temporal sequence of the concurrent assembly of two different types of spindles. The involvement of MTOCs may have facilitated the rapid constitution of bipolar spindles, whereas anastral bipolar spindle assembly, which depends solely on microtubule self-organization around chromosomes, may be a slower process, as has been shown in *D*. *melanogaster* somatic cells (Basto et al. 2006; Lecland et al. 2013; Poulton et al. 2014).

It was curious that we failed to detect embryos with biastral and anastral bipolar spindles during the second mitosis. Instead, as category 4 embryos showed, biastral and monastral bipolar spindles were distributed independently (Fig. 5d). It is thus highly likely that anastral spindle poles strongly attract a nearby aster. One of the poles of an anastral spindle may often be caught by astral microtubules that emanate from the neighboring biastral spindle, resulting in the dual spindle in category 2 (27.1%; Fig. 5b), and, less frequently, by aster emanating from an adjacent free MTOC, resulting in a monastral spindle (category 4, 6.8%; Fig. 5d). This prediction was supported by the presence of dual spindles associated with asters at both distal poles, which occurred infrequently (category 5, 3.4%; Fig. 5e). The form might result from both binding of a resident MTOC of the neighboring biastral spindle to one of the poles of the originally anastral spindle and addition of a free MTOC to another anastral pole. Taken together, our observations illustrate the plasticity of the arrangement of two spindles in the syncytium and reveal the role of asters as an “inter-spindle linker.”

In the distinctive configurations of spindles represented by categories 2 and 5, we were observing true second mitotic spindles rather than meiosis II spindles of structural similarity. Meiotic spindles arrayed in tandem lack asters at both distal poles and contain a non-centrosomal aster only between the central poles (Fig. 2, a and b; Riparbelli and Callaini 1996; Endow and Komma 1998; Wilson and Borisy 1998; Riparbelli and Callaini 2005). Instead, the asters on the dual spindles in parthenogenetic embryos were attached to both central and distal poles, and, moreover, the mitotic asters grew out from MTOCs with Asl labeling (Fig. 5, b and e). Thus, free MTOCs produced de novo in the egg cytosol did not gain immediate access to meiotic spindles, but rather exerted an impact on development during the first cleavage division. Despite the disparate architecture of these MTOCs located at the central poles of meiotic and mitotic spindles, they may share the common property of arranging two spindles in tandem. This is remarkable given that, during anaphase II, separated chromatids migrate to the central poles from discrete metaphase plates of the spindles and then the non-daughter nuclei come into close apposition (Riparbelli and Callaini 1996; Endow and Komma 1997).

### Possible microtubule interactions between anastral spindle poles and astral microtubules emanating from separate MTOCs

In post-meiotic parthenogenetic embryos, microtubules nucleate in the vicinity of a meiotic product and become organized into a bipolar array, depending on centrosome-independent mechanisms (Duncan and Wakefield 2011; Petry 2016). However, mitotic progression into anaphase was permitted only with astral bipolar spindles, which formed quite frequently during the first mitosis of parthenogenetic embryos (>70%; Fig. 3e). We thus infer that a high affinity of interaction between the aster emanating from a free MTOC and an anastral mitotic spindle might have resulted in such a conjoined structure, but this has not been demonstrated directly. It has been shown that a significant proportion of spindle microtubules are disconnected from the centrosome within a normal astral spindle (Merdes and Cleveland 1997; Chavali et al. 2015; Martin and Akhmanova 2018). In *D*. *melanogaster*, the microtubule-based processive motor dynein is required to maintain the attachment of centrosomes to mitotic spindle poles in early embryos (Robinson et al. 1999) and Schneider 2 cultured cells (Maiato et al. 2004; Goshima et al. 2005; Morales-Mulia and Scholey 2005).

According to a model for pole focusing of astral spindles (Goshima et al. 2005), temporal cross-linking between kinetochore fibers and astral microtubules by the non-clarlet disjunctional protein facilitates recruitment of dynein for minus end–directed transport of kinetochore fibers along astral microtubules toward the centrosome, forming parallel filament overlapping. Attachment of astral microtubules to separate spindle microtubules might be an integral part of astral spindle formation during the first mitosis of parthenogenetic embryos of *D*. *ananassae*. It would be an intriguing possibility that this process depends on parallel microtubule interactions involving dynein. The concurrent presence of meiotic products surrounded by microtubules and free MTOCs nucleating asters in a limited area of parthenogenetic embryos (Fig. 2c) could have increased the accessibility of astral microtubules to the self-assembled anastral spindle. It may be that initial microtubule interaction occurs most frequently during early steps of anastral spindle assembly with dispersed spindle microtubule minus ends (Supplementary Fig. 2b).

In parthenogenetic embryos, the first mitosis on a monastral bipolar spindle was reflected in the construction of one astral and one anastral spindle in the near distance during the second cycle, each encompassing replicated chromosomes of one of the daughter nuclei. Again, as discussed above for the first mitosis, it is tempting to speculate about a dominant influence of MTOCs on dual spindle formation. Presumably, astral microtubules emanating from MTOCs, which are located in the cytoplasm or attached to a bipolar biastral spindle, interact with microtubule minus ends of a forming or completed anastral spindle. Association of MTOCs to anastral spindle poles may depend on the orientation of the divisions and distribution of free MTOCs in the syncytium. Perhaps dual spindles, which are arranged in tandem with a shared MTOC (Fig. 5, b, e, and f), result from interaction between astral microtubules emanating from a spindle pole-located MTOC and microtubule minus ends of coexisting anastral spindle. Likewise, asters of free MTOCs could interact with the remaining anastral pole of dual spindles (Fig. 5e), or either one (Fig. 5d) or both (Fig. 5a) of the poles of an independent anastral spindle. During normal development of *D*. *melanogaster* early embryos, astral microtubules of biastral bipolar spindles act to prevent abnormal fusion of spindles and collision of cleavage nuclei (Baker et al. 1993). A recent report shows that, in telophase of syncytial nuclear divisions, distance between non-daughter nuclei is maintained by an aster-mediated repulsion, where antiparallel microtubule interactions are involved in a mechanical link between astral microtubules extending from neighboring centrosomes of two separate spindles (Deshpande et al. 2022). In contrast, particularly when centrosomes are defective in early embryos, abnormal connections between poles of distinct spindles located in proximity are often caused by sharing an MTOC, resulting in a configuration referred to as trains or rosettes (Megraw et al. 1999; Vaizel-Ohayon and Schejter 1999; Pérez-Mongiovi et al. 2005; Archambault et al. 2007; Zhang and Megraw 2007).

### Possible routes of diploidization for haploid eggs during parthenogenetic development in *D. ananassae*

Once dual spindles formed in parthenogenetic embryos, the structure was steadily maintained and was the means by which chromosome segregation occurred (Fig. 5b). Although the consequence of the second mitosis with dual spindles and the subsequent development were of great interest to us in the present study, this analysis was hampered, largely because of the difficulty in identifying corresponding embryos in fixed samples. In fact, during the second mitosis, asynchronous mitotic progression with two spindles, one in metaphase and the other in anaphase or telophase, was evident in 16.9% of the embryos (*n* = 2/30 in category 1, *n* = 6/16 in category 2, *n* = 1/6 in category 3, and *n* = 1/4 in category 4). As expected, we observed embryos carrying odd-numbered spindles (e.g. three spindles in an embryo as shown in Fig. 2e). Moreover, as nuclear cycles continued in parthenogenetic embryos, aberrant mitotic figures became apparent. Small thin bipolar arrays of microtubules associated with a subset of chromosomes were arranged radially around one MTOC (on the left side of Supplementary Fig. 1c). Also present were tripolar spindles (on the center right side of Supplementary Fig. 1c) and irregular microtubule arrays around dispersed chromatin involving supernumerary MTOCs (Supplementary Fig. 1d). Besides the formation of wholly haploid embryos (Supplementary Fig. 1b), such defects in spindle assembly would also be responsible for many of the observed mortality of parthenogenetic embryos (Table 1).

**Fig. 6.**
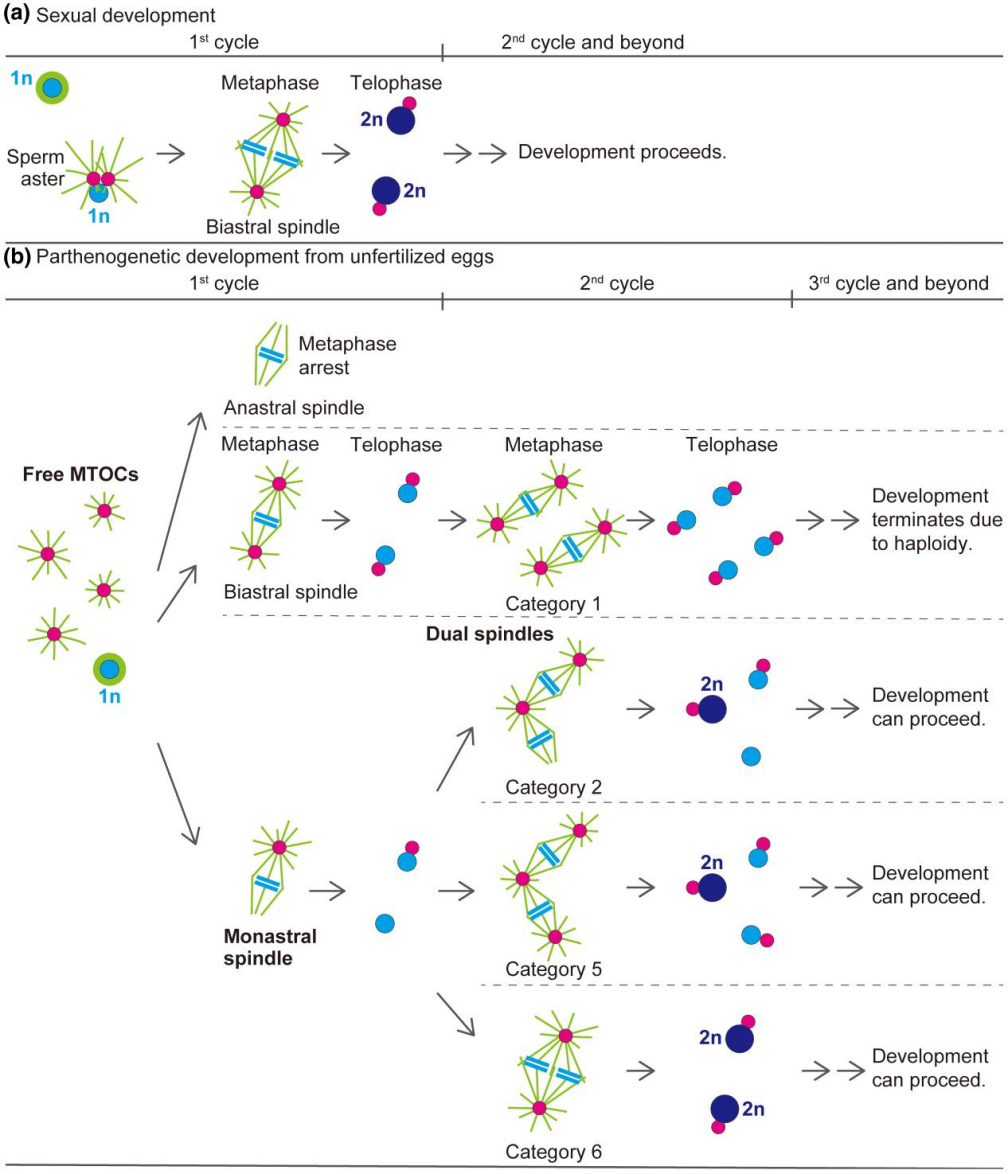
A model for diploid nucleus formation in parthenogenetic embryos of *D. ananassae*. a) On the first mitotic spindle in fertilized eggs produced by the sexual strains, which consists of a biastral bipolar spindle, parental sets of replicated chromosomes are separately surrounded by two microtubule units. The zygotic diploid (2n) nuclei are generated by fusion of parental sets of separated chromatids during telophase at the spindle poles. b) Unfertilized parthenogenetic embryos are potentially capable of generating a 2n nucleus late during the second mitosis. In unfertilized eggs produced by the parthenogenetic strain, meiosis is completed and chromosomes of meiotic products are replicated. Concurrently, free MTOCs are formed de novo in the egg cytosol. A bipolar spindle is self-assembled around a haploid (1n) set of replicated chromosomes. Depending on the absence or presence of MTOCs that were embedded in the spindle, the first mitotic spindle becomes either anastral, biastral, or monastral. With anastral bipolar spindles, mitosis is terminally arrested in a metaphase-like state. With biastral bipolar spindles, mitotic progression occurs. A series of nuclear divisions follows, resulting in embryonic lethality due to haploidy. The first mitosis is completed even with monastral spindles, resulting in 1n daughter nuclei, one is associated with an MTOC but the other is not. This apparently imperfect situation seems likely to allow parthenogenetic embryos to generate a 2n nucleus by forming dual spindles during the second mitosis. The indicated categories of the mitotic configurations of the second cycle are from Fig. 5. Category 5 involves additional attachment of a free MTOC to an anastral spindle pole during the second mitosis. Using bipolar spindles that are arranged in tandem (categories 2 and 5) or in parallel (category 6), diploidization occurs by the gathering of two sets of separated chromatids late during the second mitosis, in a fashion similar to that which occurs in fertilized eggs as depicted in (a). Categories 3 and 4 are not shown here because they could potentially represent intermediate states in mitotic organization to form dual spindles classified in categories 2 and 5, respectively.

Although achieving diploidy during parthenogenesis in *Drosophila* is generally considered to involve fusion of haploid nuclei (Templeton 1983), we do not know exactly how it occurs in parthenogenetic embryos of *D*. *ananassae*. However, the observations presented here lead us to hypothesize that the tandemly aligned configuration of dual spindles could explain a mechanistic basis for moving haploid sets of chromosomes for fusion. Figure 6 shows a model for diploidization in parthenogenetic embryos in comparison with that in sexual embryos. It is intriguing that, regarding the mechanism of diploidization, dual spindles that form during the second mitosis of parthenogenetic embryos are essentially analogous to the first mitotic spindle of fertilized eggs in sexual strains with two parental sets of chromosomes. In fertilized eggs, diploidization occurs by the gathering of two haploid sets of separated chromatids at both poles of the first mitotic spindle (Figs. 4a and 6a). Such a fusion between non-daughter nuclei could also occur late during the second mitosis of parthenogenetic embryos, at the connected poles of dual spindles (Figs. 5, b, e, f and 6b). Importantly, those resulting diploid nuclei in parthenogenetic embryos are associated with an MTOC, ensuring subsequent proliferation as nuclear cycles continue. The remaining haploid sets of chromosomes that might have migrated to the distal poles may fall out or be outcompeted by the diploid lineage thereafter in the syncytium. The probable diploidization mechanism involving fusion of haploid cleavage nuclei can account for the complete genetic homozygosity of parthenogenetic individuals of this species (Futch 1972, 1973; Matsuda and Tobari 2004).

Overall, in *D*. *ananassae*, de novo formation of free MTOCs in the egg cytosol primarily distinguished embryos produced by females of the parthenogenetic strain from those of the sexual strains. The behavior of the asters formed from MTOCs apparently holds the key to the execution of parthenogenesis. Although the participation of such MTOCs in a bipolar spindle that self-assembles around a haploid set of chromosomes leads to mitotic progression, it is considered critical that no more than one MTOC is involved during the first mitosis so that dual spindles form during the subsequent (i.e. second) mitosis (Fig. 6b). We thus suggest that parthenogenesis with a low probability of success in *D*. *ananassae* is primarily attributable to the variability in the number of MTOCs and their initial distribution within the egg. Realization of a monastral mitotic spindle for the first mitosis probably depends on interactions between anastral spindle poles and asters nucleated by free MTOCs. It is just conceivable that successful editing of parthenogenetic embryos so as to reduce the number of de novo–formed MTOCs to about one and setting it next to the meiotic product farthest from the cortex could enhance the probability of monastral spindle formation during the first mitosis and, eventually, the efficiency of parthenogenetic success.

Unlike the process in *D*. *ananassae*, parthenogenesis of unfertilized embryos of *D*. *mercatorum* occurs via an anastral bipolar spindle that is self-assembled during the first haploid mitosis and functions in an orderly way, resulting in the production of daughter nuclei lacking MTOCs (Riparbelli and Callaini 2003, 2008; Eisman and Kaufman 2007). Embryos of *Sciara coprophila* undergo a similar process (de Saint Phalle and Sullivan 1998). Thus, the role of centrosomes and the regulation of mitotic progression during syncytial cleavage divisions can differ between species. Nevertheless, regarding the diploidization mechanism of parthenogenetic embryos, centrosomes are assumed to be involved in capturing two of the haploid cleavage nuclei distributed in early embryos of *D*. *mercatorum* (Eisman and Kaufman 2007), as we observed in the second mitosis of *D*. *ananassae* embryos. These findings thus underscore the important role of centrosomes in coordinating diploidization of haploid nuclei at the beginning of sexual, as well as parthenogenetic, development.

Unfertilized eggs of *D*. *melanogaster*, which are arrested in a mitotic state, normally lack maternal PCM and centrioles and also the ability to form them de novo. However, numerous free MTOCs can be produced by overexpression of five different centrosomal proteins (Spindle assembly abnormal-4, Spindle assembly abnormal-6, Sak kinase/Polo-like kinase 4, Anastral spindle 2, and Asl) individually in the female germline (Peel et al. 2007; Rodrigues-Martins et al. 2007; Stevens et al. 2010) or by a dominant-negative allele of the *Dhc64C* cytoplasmic dynein heavy chain gene (Belecz et al. 2001), and maternal centrosomes can be retained throughout oogenesis by preventing normal loss of centriolar Polo kinase (Pimenta-Marques et al. 2016), albeit with no resulting parthenogenetic offspring in any of these cases. Among a number of proteins that localize to the centrosome in *Drosophila*, only the above-mentioned five proteins are essential for centriole assembly and three proteins (Polo, Cnn, and Spindle defective 2) for mitotic PCM assembly (Wainman 2021). Although the exact function of these key components and their interactions to form the centrosome remain to be fully elucidated, recent studies on de novo formation of MTOCs provide new insights into the pathways of centriole and centrosome assembly. Aggregated PCM proteins serve as a scaffold in the cytoplasm and accumulate proteins necessary for centriole de novo assembly (Nabais et al. 2021). Self-organization of cartwheel proteins can be used to probe centriole and mitotic PCM assembly (Gartenmann et al. 2020).

In females of the parthenogenetic strain of *D*. *ananassae*, elimination of maternal centrosomes occurred normally prior to meiosis (Fig. 1g). It is thus possible that maternal control of centrosome biogenesis is altered and an MTOC self-organizing pathway becomes operational upon egg activation, providing a strong likelihood of parthenogenesis initiation. The expression of any centrosomal genes in oocytes could be high enough to transiently induce de novo formation of MTOCs at the anterior of the egg, or, alternatively, a normal mechanism to suppress their de novo formation could be lost. It would be interesting in the future to understand the basis of the maternal effect by which de novo formation of MTOCs can be induced.

## Supplementary Material

iyac178_Supplementary_Data

## Data Availability

Strains are available upon request. The authors affirm that all data necessary for confirming the conclusions of the article are presented within the article, figures, and tables. More information can be found in the Supplemental material. Supplemental material available at *GENETICS* online.

## Notes

*Communicating editor: B. Calvi*
