# Supplementary material for: Mitotic progression and dual spindle formation caused by spindle association of de novo–formed microtubule-organizing centers in parthenogenetic embryos of Drosophila ananassae
Source: Genetics. 2022 Dec 14;223(2):iyac178. doi: 10.1093/genetics/iyac178 (PMC9910410; doi:10.1093/genetics/iyac178)
Supplement: iyac178_Supplementary_Data [file iyac178_supplementary_data.zip › Supplemental_Material_Legends_GENETICS-2022-305745.docx]

**Supplemental Figure Legends**

**Figure S1. Nuclear divisions in syncytial embryos produced by females of the sexual and parthenogenetic strains.**

Confocal images of 3–6 hr-old syncytial embryos treated with antibodies against α-Tubulin (αTub; green in merged images) for microtubules and against Centrosomin (Cnn; magenta in merged images) and Asterless (Asl; gray; not overlaid in merged images but only shown independently) for the PCM and with the DNA dye DAPI (light blue in merged images). (A and B) Embryos with bipolar spindles showing synchronous cleavage divisions. (A) Diploid mitoses in sexually developing embryos at the syncytial blastoderm stage. (B) Haploid mitoses in unfertilized parthenogenetic embryos at the syncytial blastoderm stage. (C and D) In the syncytium of parthenogenetic embryos, scattered DNA and non-bipolar microtubule arrays were often observed. (C) The embryo contains tripolar spindle (on the center right side), radially arranged, bipolar arrays of microtubules (on the left side), and a bipolar biastral spindle (on the upper right side). (D) A large tubulin aggregates are associated with a number of MTOCs, where the majority of chromosomes appear aligned at the center whereas the remaining ones spread out within the structure (on the left side). Microtubules form around the four MTOCs, and bipolar arrays of microtubules also self-assemble with a subset of chromosomes (on the right side). Scale bars, 10 μm.

**Figure S2. Meiosis and the first mitosis in unfertilized embryos produced by females of the parthenogenetic strain.**

Confocal images of fixed embryos labeled with antibodies against α-Tubulin (αTub; green in merged images) for microtubules and against Centrosomin (Cnn; magenta in merged images) and Asterless (Asl; gray; not overlaid in merged images but only shown independently) for the PCM and with the DNA dye DAPI (light blue in merged images). (A) Apparently normal anaphase I in the presence of free microtubule-organizing centers in the cytosol. (B and C) Mitotic figures in the first mitosis of unfertilized parthenogenetic embryos. The small microtubule arrays are self-assembled around individual or a subset of chromosomes, independently of the asters that emanate from MTOCs. Scale bars, 10 μm.
