# Supplementary figures and images for: Mitotic progression and dual spindle formation caused by spindle association of de novo–formed microtubule-organizing centers in parthenogenetic embryos of Drosophila ananassae
Source: Genetics. 2022 Dec 14;223(2):iyac178. doi: 10.1093/genetics/iyac178 (PMC9910410; doi:10.1093/genetics/iyac178)

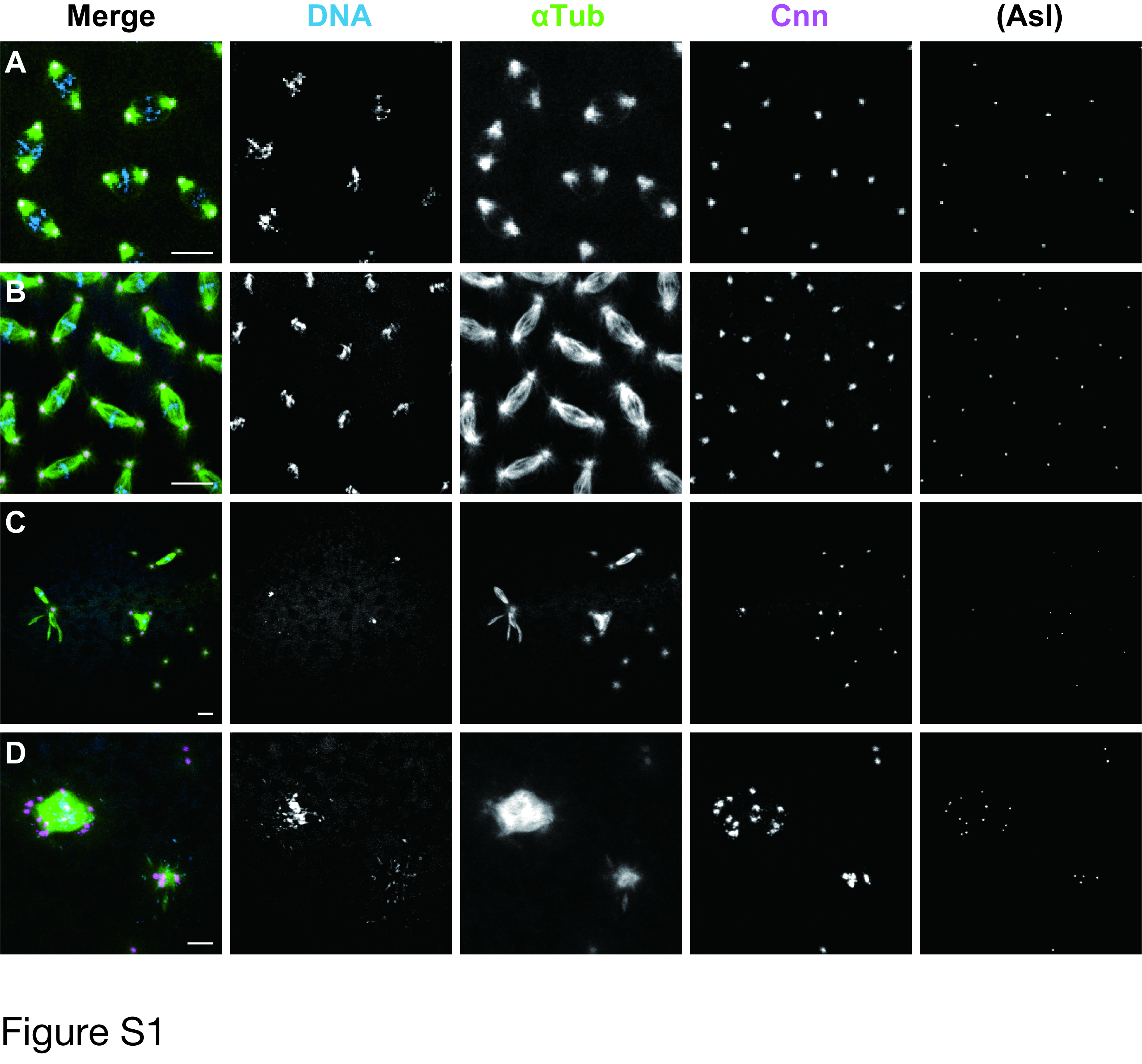

Supplement: iyac178_Supplementary_Data [file iyac178_supplementary_data.zip › Figure_S1_GENETICS-2022-305745.tif]

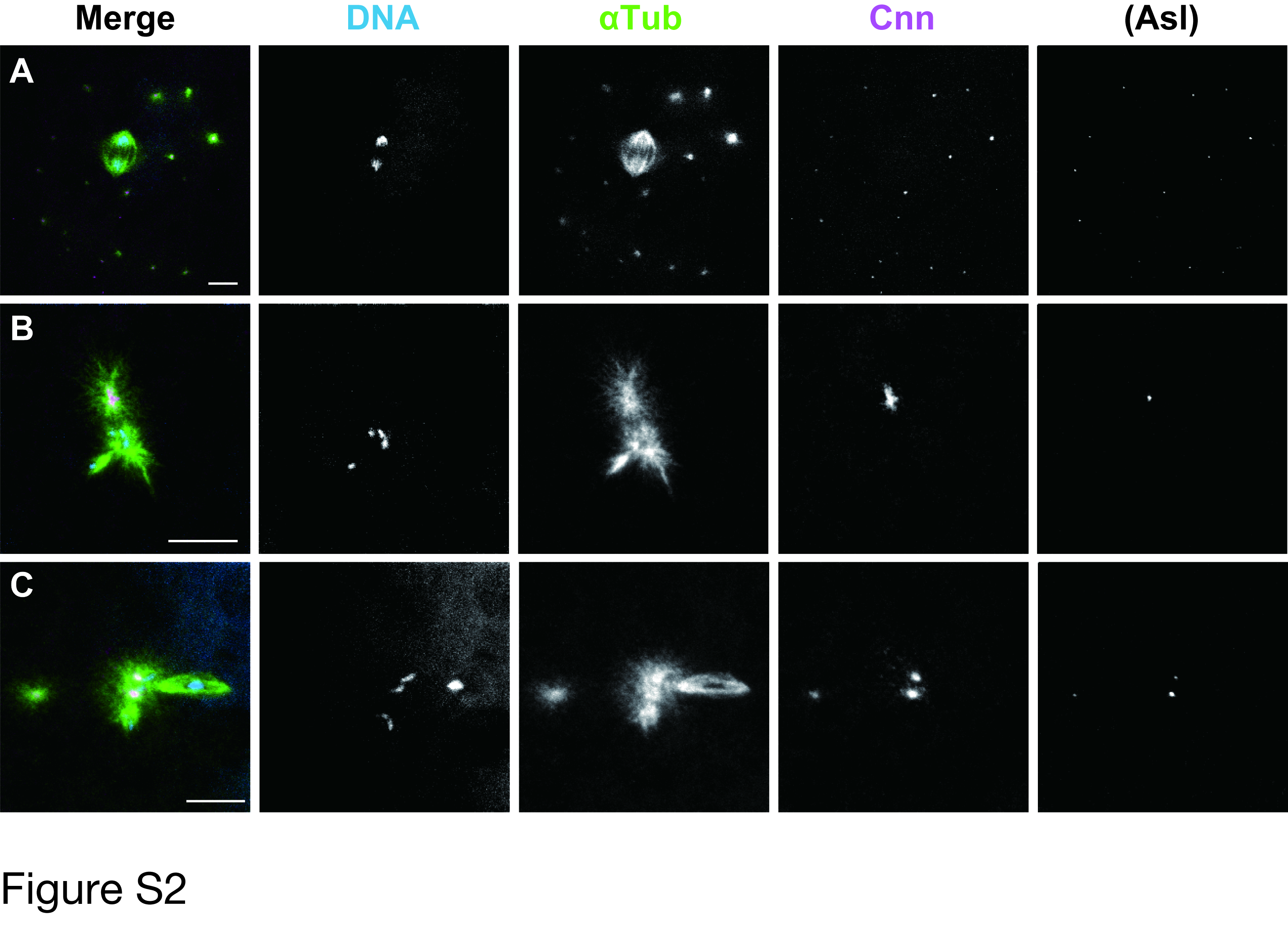

Supplement: iyac178_Supplementary_Data [file iyac178_supplementary_data.zip › Figure_S2_GENETICS-2022-305745.tif]
